# A novel nomogram for predicting risk of malnutrition in patients with heart failure

**DOI:** 10.3389/fcvm.2023.1162035

**Published:** 2023-03-23

**Authors:** Jian Liu, Shengjia Xu, Jiurui Wang, Jing Liu, Zeping Yan, Qian Liang, Xiaorong Luan

**Affiliations:** ^1^ School of Nursing and Rehabilitation, Cheeloo College of Medicine, Shandong University, Jinan, China; ^2^ The First Clinical College, Shandong University, Jinan, China; ^3^ University of Health and Rehabilitation Sciences, Qingdao, China; ^4^ School of Nursing and Rehabilitation, Shandong University/Department of Infection Control, Qilu Hospital, Shandong University, Jinan, China

**Keywords:** heart failure, malnutrition, nomogram, China, prevention

## Abstract

**Background and aims:**

This study aimed to explore the risk factors of malnutrition in patients with heart failure and construct a novel nomogram model.

**Methods and results:**

A cross-sectional study based on the STROBE checklist. Patients with heart failure from July 2020 to August 2021 were included. Patients were divided into a malnutrition group and a normal nutrition group based on the Society's recommended AND-ASPEN standard. Logistic regression was used to analyze the independent risk factors for malnutrition. A new prediction model of nomogram was constructed based on the risk factors, and its fit and prediction performance were evaluated. Of 433 patients, 66 (15.2%) had malnutrition and 367 (84.8%) had normal nutrition, Logistic regression analyses showed that the risk factors for malnutrition were total protein, hemoglobin, triglyceride, and glucose levels. The regression model based on the above four variables showed an area under the curve of 0.858. The novel nomogram model had a sensitivity of 78.5% and a specificity of 77.3%. After 2000 bootstrap resampling iterations, AUC was 0.852.

**Conclusions:**

The novel nomogram model can predict the odds of malnutrition in patients with heart failure at the early stage of admission, and can provide a reference for nursing staff to optimize nutritional care for inpatient with heart failure and to develop a discharge nutritional care plan.

## Highlights

• This is the first study to construct a novel nomogram model for predicting the odds of malnutrition among patients with heart failure in China.
• Total protein, hemoglobin, triglyceride, and glucose are independent risk factors for malnutrition in patients with heart failure.
• The model has a good fitting effect and discriminatory ability.
• The nomogram model can help clinicians and nurses to predict the odds of malnutrition in patients with heart failure admitted to hospital, and it is helpful to optimize nursing strategies.

## Introduction

Heart failure (HF) is considered an epidemic today, with more than 64 million cases of HF worldwide (1). The prevalence of HF is expected to increase by 46% by 2030 (2). In addition, patients with HF are characterized by high readmission and mortality rates (3, 4).

Malnutrition is a disease in which the intake of various nutrients is insufficient or imbalanced, leading to a decrease in body function and a deterioration in clinical outcomes (5). Malnutrition and severe alteration of the protein components of the body (protein disarrangements) are common conditions in patients with HF. A meta-analysis showed that the prevalence of malnutrition in patients with HF ranged from 16 to 90% (6). HF related malnutrition may be caused by the following mechanisms: low nutritional in-take due to intestinal edema and anorexia (7), hepatic dysfunction (8), cytokine-induced hyper catabolism (9), and insulin resistance(10).

Disease-related malnutrition significantly increases medical costs, and its importance in the course of the disease remains underappreciated (11). The malnutrition status in patients with HF is undoubtedly related to the quality of life, risk of re-hospitalization, prolonged hospitalization, complications, and increased risk of death during in-patient treatment (12, 13). Studies have confirmed that malnutrition is independently correlated with heart dysfunction, disease progression, and mortality, and it can lead to a series of negative consequences (14). Okoshi et al. found that malnourished hospitalized patients with HF had a worse prognosis than patients with normal nutritional status, and that malnutrition was significantly associated with increased readmission and mortality (15). In addition, malnutrition may also be a driver of disease progression as part of a vicious cycle associated with cytokine activation, autonomic dysfunction, and cachexia, leading patients with HF into enter a vicious cycle of malnutrition-inflammatory response-cachexia (16, 17). The most severe form of malnutrition in patients with HF is cardiac cachexia, a state of catabolic failure associated with inflammation and neurohormonal activation that can severely reduce the survival rate of patients with HF, and once a patient enters the cardiac cachexia stage, it is difficult to reverse the disease process even with treatments such as nutritional support (18, 19). Malnutrition also imposes a significant financial burden on patients with HF and health care organizations. Studies have shown that the average cost to the health care system for patients with HF diagnosed with malnutrition is twice than that of non-malnourished patients (20).

Malnutrition is a modifiable risk factor that clinicians and caregivers can act on (21). As the primary manager of nutritional care for patients with HF in the early stages of hospital admission, early identification of risks associated with malnutrition and interventions are critical. In addition, nutritional interventions can improve cardiopulmonary function and quality of life in patients with HF, thereby reducing mortality and risk of readmission (22). Therefore, early nutritional assessment, identification of malnutrition risk, and implementation of interventions are key components to prevent disease progression and improve poor outcomes in patients with HF.

Nutritional assessment to determine the nutritional status or nutritional risk of patients with heart failure is the first step in nutritional management. Current guidelines recommend timely assessment of the nutritional status of patients with HF (23). This study identifies malnutrition and normal nutrition patients for this study based on the definition of malnutrition by the Academy of Nutrition and the American Society for Parenteral and Enteral Nutrition, and on the AND-ASPEN criteria recommended by the Academy (24). Some studies have shown that this criterion is valid compared to with the subjective global assessment (SGA) (25). However, currently, the importance of on nutritional risk in patients with HF is often ignored. In fact, it is believed that malnutrition is currently underdiagnosed and therefore undertreated, resulting in a high prevalence of malnutrition in patients with HF. Early detection of metabolic abnormalities in malnutrition individuals with HF may have important public health implications. A first systematic review of blood biomarkers of malnutrition in adults found that some blood biochemical markers, such as hemoglobin, and total cholesterol, are useful biomarkers of malnutrition in adults (26). However, there are few studies on screening for nutritional risk in patients with HF, and specific tools are lacking. Therefore, it is important to explore a clinically applicable tool to predict the odds of malnutrition in patients with HF.

Medical nomogram prediction models are used to describe a statistical prognostic model using biological and clinical variables, which allow the model to be presented in a graphical manner (27). The nomogram-based clinical prediction model can help clinical healthcare professionals to calculate the odds of adverse risk for each patient conveniently, quickly, and efficiently, to better adapt to the cumbersome clinical environment, and to facilitate clinical dissemination and application (28–30). Therefore, this study aimed to construct a nomogram-based malnutrition risk prediction model by analyzing the factors influencing malnutrition in patients with HF to provide clinicians and caregivers with a malnutrition screening tool for patients with HF to help develop medical care plans and optimize care strategies.

## Methods

### Subjects and design

The study was a single-center cross-sectional study. Our cases were from patients who admitted to the department of cardiology in a hospital in Shandong, China from July 2020 to August 2021.

The inclusion criteria were as follows: (1) being older than 18 years, (2) HF was diagnosed according to the recommendations of the Chinese guidelines for the diagnosis and treatment of HF (31), (3) Patients with heart failure in NYHA class II-IV. The exclusion criteria were as follows: (1) severe chronic liver or kidney disease, (2) autoimmune or chronic inflammatory disease, (3) severe cognitive impairment such as Alzheimer's disease and psychiatric disorders, (4) Patients with acute heart failure due to other causes such as acute myocardial infarction.

### Data collection

In this study, the purpose of the study and informed consent were explained to patients at the time of admission, and basic patient information was collected by questionnaire within 24 h after admission, while the first clinical laboratory indexes were collected in the hospital electronic case system, including (1) basic conditions: age, gender, New York Heart Association (NYHA), BMI; (2) disease conditions: heart rate, etiology of HF, the Charlson comorbidity index (CCI); (3) laboratory tests: serum albumin, glucose, NT-proBNP, serum sodium, hemoglobin, cholesterol, lymphocyte count, etc.; (4) ancillary tests: electrocardiogram, echocardiogram. All data were entered by 2 researchers, and the data set was validated and cleaned to prevent any further changes before statistical analysis was performed.

This study complied with the Declaration of Helsinki and was approved by the research ethics committee of hospital. Informed consent was obtained from all the participants prior to any study-related activities.

### Definitions and criteria of related indicators

Body mass index (BMI) was calculated for all patients, defined as the body mass (in kilograms) divided by the square of the body height (in meters) (32).

Charlson Comorbidity Index (CCI), a weighted index that explains the presence of 17 co-morbidities (33).

### Outcome

This study identifies malnutrition patients based on the AND-ASPEN criteria recommended by the Society, which include energy intake, weight loss, and physical findings from a nutrition focused physical examination (NFPE). We defined nutritionally normal as patients who did not meet the criteria for moderate or severe malnutrition, and malnutrition patients as those who met the criteria for moderate or severe malnutrition.

### Data analysis

All the statistical analyses were performed using IBM SPSS/WIN version 26.0 and R 4.1.1. Measures conforming to a normal distribution were expressed as [mean ± SD], and group comparisons were made using Independent Student's t-test; Measures that did not conform to a normal distribution were expressed as median [M (P25, P75)], and the Mann-Whitney *U* test was used for comparison between groups; categorical data are expressed as [*n* (%)], The chi-square test was used to compare proportions between groups.

The tolerance and variance inflation factor (VIF) were used to analyze the multicollinearity of the samples, and all the variables in this study had VIF <5, which is considered to indicate a lack of collinearity problem between the variables. Univariate logistic regression was used to examine the relationship between each risk factor and the nutritional normal group and the malnutrition group. Variables that were significant in the univariate analysis were included in the multifactorial logistic regression model. Models were constructed based on the results of multifactorial logistic regression to facilitate the use of the nomogram in clinical practice. The nomogram-based risk prediction model was established using R4.1.1 software. Model performance was validated by the area under the receiver operating characteristic curve (AUC), Hosmer-Lemeshow statistic, sensitivity, and specificity. The closer these metrics are to 1, the better is the model performance. A 2000-fold bootstrap resampling method was used for internal validation of results. The AUC was used to test the performance of the prediction model. The model was assessed for clinical utility using decision curve analysis (DCA). All *P* values were two sided, and values <0.05 were considered significant.

## Results

### Demographic data and univariate analysis results

A total of 433 patients with HF were enrolled in this study. Most patients in the study population were male and elderly, with a mean age of 62.09 ± 13.65 years, of which 66.1% were male. The median NT-proBNP was 5,229 (48-63990) ng/L. More than 70% of patients had severe symptoms (NYHA class III/IV). 66 (15.2%) of the 433 patients had malnutrition and 367 (84.8%) were nutritionally normal. There was a statistically significant difference between the malnutrition group and the normal nutrition group in terms of etiology of HF, CCI, and type of admission (*P *< 0.05). The differences in age, gender, sedentary hours, smoking history, drinking history, and NHYA classification were not statistically significant (all *P*s > 0.05) (Table 1).

**Table 1:** Univariate analysis of the malnutrition in patients with heart failure.

Predictor	HF patients	Normal nutrition	Malnutrition	*χ*2/*t/Z*	*P* value
	(*n* = 433)	(*n* = 367,84.8%)	(*n* = 66,15.2%)		
Age, years	62.09 ± 13.65	61.97 ± 13.86	62.76 ± 12.46	−0.433	0.665
Sex				0.536	0.464
Men	286 (66.1)	245 (66.8)	41 (62.1)		
Women	147 (33.9)	122 (33.2)	25 (37.9)		
Sedentary				1.418	0.234
<6 h	239 (55.2)	207 (56.4)	32 (48.5)		
≥6 h	194 (44.8)	160 (43.6)	34 (51.5)		
Etiology				6.075	0.048
Coronary heart disease	233 (53.8)	190 (51.8)	43 (65.2)		
Dilated cardiomyopathy	74 (17.1)	69 (18.8)	5 (7.6)		
Other types of cardiomyopathies	126 (29.1)	108 (29.4)	18 (27.3)		
CCI	2.38 ± 1.32	2.29 ± 1.27	2.89 ± 1.50	−3.085	0.003
NHYA classification				3.268	0.195
II	116 (26.8)	103 (28.1)	13 (19.7)		
III	206 (47.6)	175 (47.7)	31 (47)		
IV	111 (25.6)	89 (24.3)	22 (33.3)		
Type of admission				11.672	0.001
Outpatient	247 (57.0)	222 (60.5)	25 (37.9)		
Emergency	186 (43.0)	145 (39.5)	41 (62.1)		
Smoking history				1.732	0.188
No	242 (55.9)	210 (57.2)	32 (48.5)		
Yes	191 (44.1)	157 (42.8)	34 (51.5)		
Drinking history				0.000	0.988
No	268 (62.0)	227 (62.0)	41 (62.1)		
Yes	164 (38.0)	139 (38.0)	25 (37.9)		
BMI	25.45 ± 4.44	25.55 ± 4.40	24.86 ± 4.65	1.158	0.247
HR	80.06 ± 18.11	79.32 ± 18.17	84.2 ± 17.35	−2.201	0.044
RDW	13.80 ± 2.13	13.69 ± 1.86	14.40 ± 3.20	−1.740	<0.001
HGB	133.08 ± 24.33	136.11 ± 22.69	116.24 ± 26.40	5.745	<0.001
LYM	1.56 ± 0.57	1.62 ± 0.56	1.24 ± 0.50	5.179	
BUN	8.48 ± 5.30	7.93 ± 4.32	11.55 ± 8.39	−3.430	0.001
TP	64.66 ± 6.07	65.78 ± 5.51	58.48 ± 5.34	9.951	<0.001
CHO	3.92 ± 1.12	3.95 ± 1.09	3.77 ± 1.27	1.222	0.222
TG	1.40 ± 0.78	1.43 ± 0.82	1.20 ± 0.45	3.253	0.001
HDL	1.01 ± 0.27	1.01 ± 0.26	0.93 ± 0.29	2.336	0.020
GLU	6.00 ± 2.33	5.83 ± 1.94	6.90 ± 3.72	−2.271	0.026
NA	141.26 ± 3.58	141.48 ± 3.39	140.02 ± 3.39	2.617	0.011
LVEF	59.06 ± 10.68	59.42 ± 10.70	57.05 ± 10.44	1.663	0.097
NT-proBNP	2,210.00 (951.25, 6,046.00)	1,990.00 (799.80, 4,820.00)	5,470.00(2,201.00, 13,800.00)	−5.368	<0.001

HR, heart rate; NYHA, New York Heart association classification; BMI, body mass index; CCI, charlson comorbidity index; RDW, red blood cell distribution width; HGB, hemoglobin; LYM, lymphocyte count; BUN, blood urea nitrogen; TP, total protein; CHO, cholesterol; TG, triglyceride; HDL, high density lipoprotein; GLU, glucose; NA, sodium; LVEF, left ventricular ejection fraction.

For the clinical characteristics and biochemical indicators during hospitalization malnutrition group compared with normal nutrition group, univariate results showed that HR (*P *= 0.044), HGB (*P *< 0.001), LYM (*P *< 0.001), BUN (*P *= 0.001), TP (*P *< 0.001), TG (*P *< 0.001), HDL (*P *= 0.020), GLU (*P *= 0.026), NA (*P *= 0.011), NT-proBNP (*P *< 0.001) may be associated with increased the odds of malnutrition. In addition, there was no statistically significant difference between the two groups for BMI, RDW, BUN, LVEF, and CTNI (*P *> 0.05) (Table 1).

### Multivariate analysis

Variables with *P *< 0.05 in the univariate analysis were included in the multivariate analysis, including etiology of HF, CCI, type of admission, admission heart rate, HGB, LYM, BUN, TP, TG, HDL, GLU, NA, and NT-proBNP. A stepwise method was used to select variables. The results of the multifactorial analysis showed that TP (OR,0.765;95% CI, 0.711–0.824), TG (OR, 0.509;95% CI, 0.304–0.852), HGB (OR, 0.979;95% CI, 0.966–0.992), GLU (OR, 1.177; 95% CI, 1.045–1.326) were an independent risk factor (Table 2). We established a risk prediction model for malnutrition on the afore-mentioned four predictors, which independently associated with the odds of malnutrition as assessed by logistic regression analysis.

**Table 2:** Multivariate logistic regression analysis for malnutrition in patients with heart failure.

Variable	*β*	SE	Wald χ2	*P*	OR (95% CI)
TP	−0.268	0.038	50.181	<0.001	0.765 (0.710–0.824)
HGB	−0.022	0.007	10.252	0.001	0.979 (0.966–0.992)
TG	−0.676	0.263	6.599	0.010	0.509 (0.304–0.852)
GLU	0.161	0.060	7.160	0.007	1.174 (1.044–1.321)
Intercept	17.429	2.463	50.068	<0.001	-

CI, confidence interval; TP, total protein; HGB, hemoglobin; TG, triglyceride; GLU, glucose; OR, odds ratio; β, regression coefficient.

### Risk prediction model

#### Model development

The risk prediction model equation is as follows, *P*(malnutrition) = e^(17.429 + −0.268 × TP + −0.022 × HGB + −0.676 × TG + 0.161 ×  GLU)^/ (1 + e^(17.429 + −0.268 × TP + −0.022 × HGB + −0.676 × TG + 0.161 × GLU)^).

#### Evaluation of the prediction model

**Figure 1:**
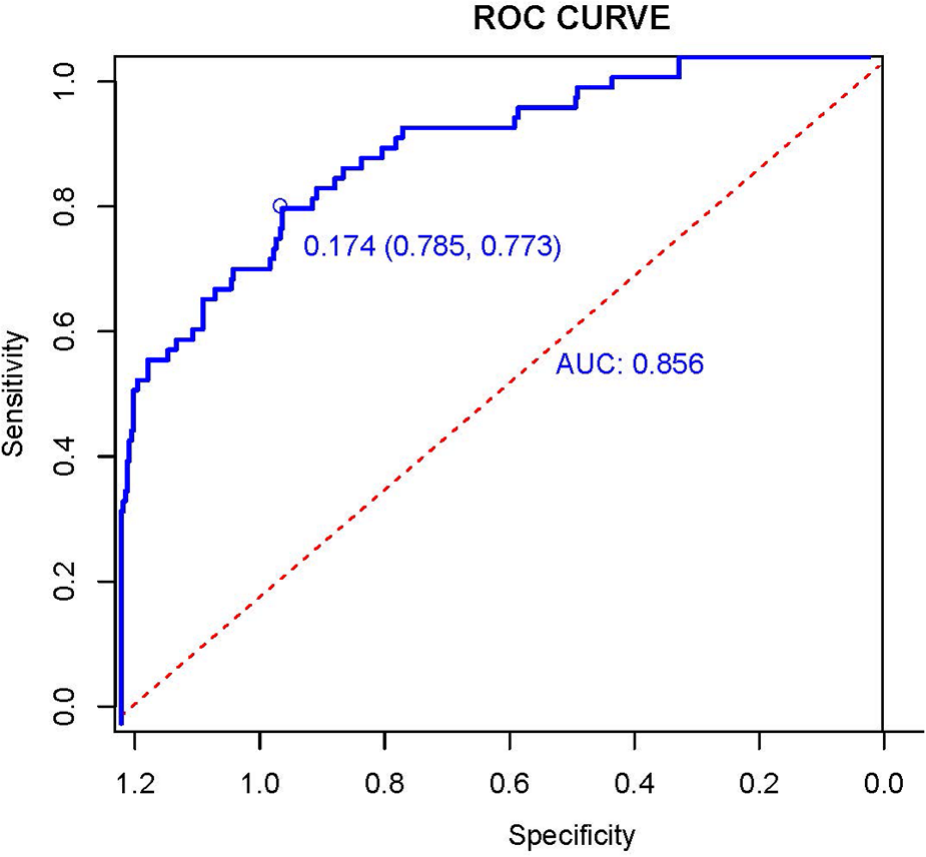
Receiver operation characteristic curve (AUC) of the logistic regression model. The AUC was used to test the performance of the prediction model.

We drew AUC, The AUC was 0.856[95% CI:0.804, 0.908; *P *< 0.001]. The results showed that the Hosmer-Lemeshow test *P *= 0.098, and the optimal cut-off value of AUC was 0.174 based on the maximum principle of Youden index, where it yielded an accuracy of 77.3%, a sensitivity of 78.5%. This indicates that the model fits better and performs better with the data of this study (Figure 1). After 2000 bootstrap resampling iterations, AUC was 0.852, which indicates that the model has good discriminatory ability.

**Figure 2:**
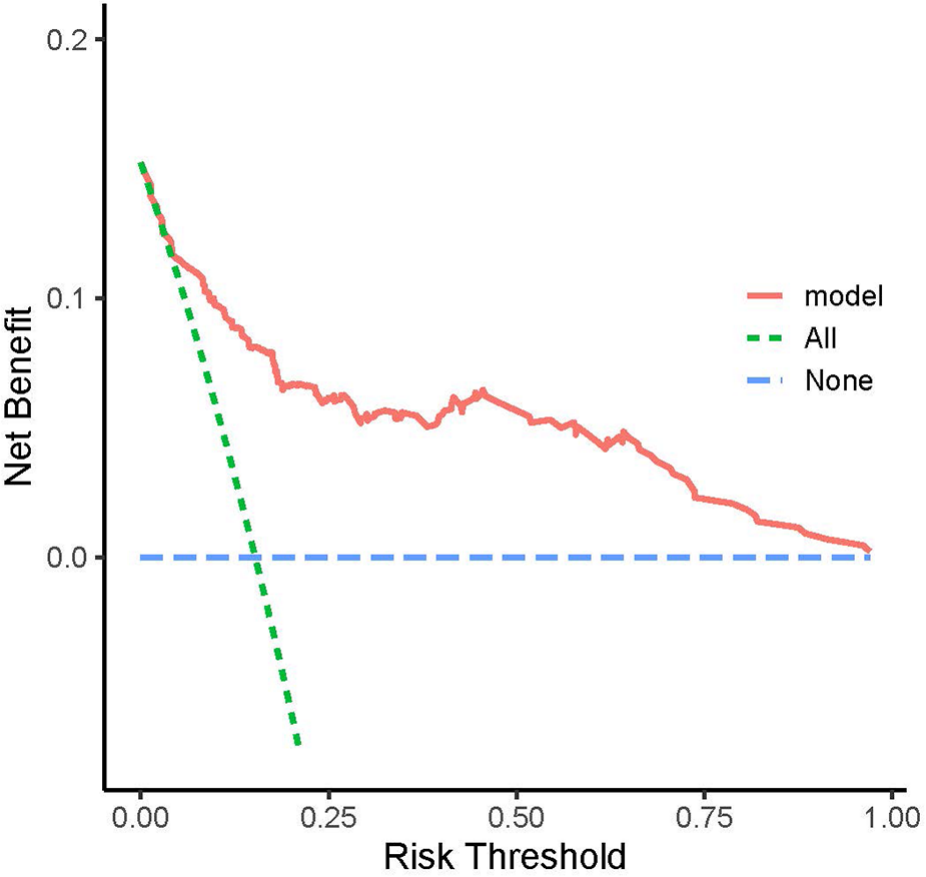
Decision curve analysis (DCA). The model was assessed for clinical utility using decision curve analysis (DCA).

The Decision curve analysis (DCA) for the model revealed that when the threshold probability of an individual was between 20% and 95%, application of this model to predict the odds of malnutrition would add net benefit than applying either the treat-all or treat-none strategies (Figure 2).

## Discussion

**Figure 3:**
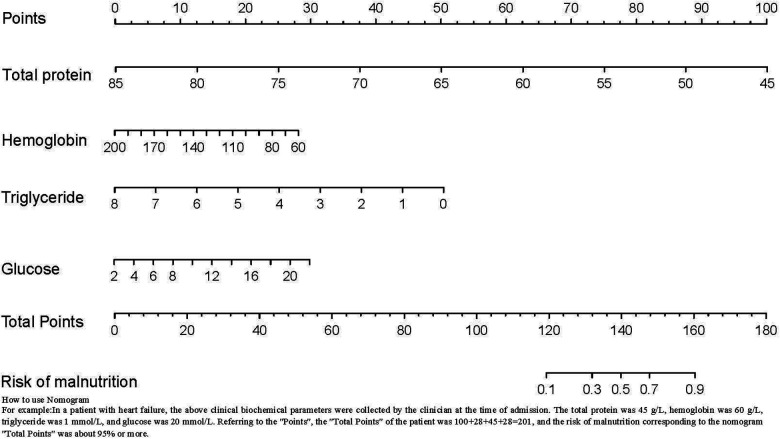
Risk prediction model for malnutrition. A risk prediction model for malnutrition in patients with HF based on risk factors was constructed and presented in the form of a line graph.

In this study, the general demographic data, and clinical laboratory biochemical indices of 433 hospitalized patients with HF were analyzed. Malnutrition and normal nutrition patients for this study were identified according to the AND-ASPEN criteria recommended by the Society. In our study, the prevalence of malnutrition was as high as 15.2%. After logistic regression analyses, total protein, hemoglobin, triglyceride, and glucose levels were found to be independent risk factors for malnutrition in patients with HF. A risk prediction model for malnutrition in patients with HF based on risk factors was constructed and presented in the form of a line graph (Figure 3). The nomogram model was shown to have good fitting and discriminatory ability and could help clinicians and nurses to predict the odds of malnutrition in hospitalized patients with HF. The best critical value of the model is 0.174. When the score is ≥0.174, it suggests that the patient is at a high odds of malnutrition, and the nursing staff should systematically assess the patient's condition and causative factors to implement targeted interventions. When the score is close to 0.174, the nursing staff need to educate the patient on importance of malnutrition, develop a prospective care plan for the patient, and provide the patient with adequate nutritional support. When the patient's condition changes between the time of hospitalization and discharge, nursing staff are advised to reassess the patient's odds of malnutrition, and adjust the nutritional care plan in a timely manner to avoid malnutrition.

This study found that for each unit of elevation in total protein, hemoglobin, and triglyceride, there was a corresponding decrease in the odds of malnutrition in patients with HF. In clinical practice, clinicians prefer the advantages provided by blood metabolic indicators in patients with HF who are malnutrition or at a the odds for malnutrition. Zhang et al. (26) conducted the first systematic evaluation of many blood biomarkers associated with malnutrition in adults to determine their relationship with malnutrition odds. They showed that albumin, hemoglobin, total cholesterol, and total serum protein, were useful biochemical markers of malnutrition. However, the study did not have sufficient evidence to include erythrocyte pressure, triglycerides, and creatinine as markers of malnutrition.

The normal range of serum total protein is usually between 6.0–8.3 g/dl, and studies have found that serum total protein levels <6 g/dl serve as a useful biomarker for malnutrition in adults (34). We did not categorize the levels of total protein and other biochemical markers in this study because we felt that using the variables as continuous variables would better reflect the true levels of these indicators in real patients. Analysis of serum albumin as a continuous variable revealed that patients with HF who had higher serum albumin levels were at a lower odds of malnutrition. This observation is consistent with a recent study of post-acute care elderly patients with lower serum total protein levels regardless of the diagnosis of malnutrition or skeletal muscle reduction (35). The mechanism underlying the association between serum albumin levels and malnutrition in patients with HF may be related to the fact that serum total protein is primarily synthesized by the liver and has functions such as nutrition, buffering and maintenance of colloid osmotic pressure (8). It has been shown that patients with HF are often at risk for malnutrition due to impaired cardiac function, leading to hemodynamic disorders, which cause abnormal liver function and lower serum total protein levels, thereby affecting the nutritional status of patients with HF (36). In addition, it has been found that dysfunction of the gastrointestinal tract has an important impact on the nutritional status of patients with HF (37). In fact, this disorder may lead to malabsorption and subsequently to malnutrition. Gut-liver system disorders are known to typically aggravate malnutrition in patients with HF (38). A systematic review indicated that total nutritional interventions can increase albumin and serum total protein levels in patients (26). Therefore, caregivers should monitor patients' serum total protein levels, administer liver and gastrointestinal medications as prescribed, and instruct patients to consume foods that are rich in high-quality proteins and easy to digest.

Normal hemoglobin range is generally defined as 13.5–17.5 g/dl for men and 12.0–15.5 g/dl for women (39), The World Health Organization defines the lower limit of normal hemoglobin for adults as (13 g/dl for men and 12 g/dl for women). Hemoglobin is often used as an important biochemical indicator of anemia. It has been shown that low albumin levels are associated with anemia and that anemic patients have an increased risk of hypoalbuminemia, which is further enhanced in malnutrition patients (40). As mentioned earlier, hypoproteinemia significantly increases the odds of malnutrition in patients. Therefore, nursing staff are prompted to pay attention to the occurrence of anemia in patients with HF during clinical care and to develop a timely and reasonable dietary nutrition plan to reduce the odds of hypoproteinemia and malnutrition. Few studies have evaluated other blood biomarkers, such as creatinine, triglycerides, iron, and red blood cell pressure in patients with HF (26). Our study found that triglyceride is a valid biological predictor of malnutrition odds in patients with HF. Cholesterol indicated in predicting the odds of malnutrition in patients with HF. Triglycerides and cholesterol are often used together as the main components of the lipid quadruple, and although few relevant studies exist, they reflect to some extent the value of this biochemical indicator in predicting the odds of malnutrition. Further investigation is needed to determine whether triglyceride is a valid indicator of malnutrition odds.

Another finding of our study was that each unit increase in glucose resulted in a 0.177-fold increase in the odds of malnutrition. There is often a relationship between glucose levels and malnutrition. Poor glucose tolerance is a feature of protein-energy malnutrition, and the prevalence of malnutrition-related gestational diabetes syndrome was found to be high in several hypothetical studies of pregnant women in the prenatal and postnatal periods. In addition to these prenatal effects, postnatal malnutrition may lead to sustained impairment of insulin secretion and glucose tolerance, leading to an increased chance of diabetes prevalence later in life (41–43). Elevated glucose levels can lead not only to an increased odds of malnutrition in patients, but also to an increased odds of HF in adults. One study found a significant association between diabetes and several cardiovascular disease risk factors (44). Blood glucose care has always been the focus of clinical nursing work, and it is particularly important to pay attention to the changes of blood glucose levels in patients with HF. Nurses should place emphasis on, carefully monitoring blood glucose levels at appropriate times. For patients with HF with large changes in blood glucose levels, timely measures should be taken to educate patients to exercise and appropriately control their diet to maintain more stable blood glucose levels and reduce the odds of malnutrition.

The traditional logistic prediction model is an equation that requires formula conversion to obtain predictive values, and nurses using such a tool in a tedious clinical environment may often miss the best time to intervene. Nomogram prediction models, which are visual presentations, allow nurses to screen patients with HF who are at a high odds of malnutrition, more easily and quickly and is more suitable in a clinical setting. For patients who are judged to be at an increased odds for malnutrition by this approach, nursing staff should develop detailed, individualized care plans to ensure that the patients receive the necessary nutrients in a timely manner to avoid causing malnutrition. In addition, although age and other variables may not be a factor in the nomogram prediction model in this study, we believe that these variables are also clinically important. We tried to include these variables, which were not statistically significant in the univariate analysis but were considered clinically significant, in the final model and found no significant increase in the predictive efficacy of the model. To present a fast and efficient tool, we decided to only include four variables. We believe that in addition to applying the model to identify patients with HF with malnutrition in a clinical setting, nurses should also pay some attention to factors such as age and co-morbidities in actual clinical work, which can provide more information for the overall health management of patients and reduce the occurrence of malnutrition in patients with HF, thus reducing the occurrence of poor prognostic outcomes.

### Limitations

The conditions of data collection in this study led to a relatively homogeneous perspective of the prediction model. However, we believe that including serum biochemicals as a part of routine blood tests to identify patients with HF at the odds for malnutrition is a quicker and more convenient means for carrying out predictions. This study was a single-center study with a limited sample size, and only internal validation of the nomogram model was needed for external validation. We will expand the sample size in future studies and prospectively explore changes in malnutrition over time in patients with HF to assess the predictive validity of the model.

## Conclusions

Total protein, hemoglobin, triglyceride, and glucose levels are independent risk factors for malnutrition in patients with HF; based on the four risk factors, a new nomogram model was constructed to predict the odds of malnutrition in patients with HF. The model demonstrated good fitting effect and discriminatory ability. The model can help clinicians and nurses to complete the screening for nutritional risk at an early stage of patient admission and predict the odds of malnutrition in patients with HF. Moreover, with our findings nursing staff can implement early interventions and precise management strategies based on these risk factors, and provide a basis for optimizing effective nursing strategy.

## Data Availability

The original contributions presented in the study are included in the article/supplementary materials, further inquiries can be directed to Jian Liu *liujian0547@163.com*.
